# Small Cell Carcinoma in the Mammary Gland: Primary or Metastatic? A Diagnostic Challenge

**DOI:** 10.4021/wjon2010.04.207w

**Published:** 2010-04-30

**Authors:** Ghazala Mehdi, Hena A. Ansari, Rana K. Sherwani, Rakesh Bhargava

**Affiliations:** a Departments of Pathology, Jawaharlal Nehru Medical College, Aligarh Muslim University, Aligarh, Uttar Pradesh, India; b Departments of Tuberculosis and Chest Diseases, Jawaharlal Nehru Medical College, Aligarh Muslim University, Aligarh, Uttar Pradesh, India

**Keywords:** Extra-mammary, Small cell neuroendocrine carcinoma, Breast metastasis, Cytological diagnosis

## Abstract

Metastatic tumours to the mammary gland are relatively uncommon as compared to primary breast malignancies. Such lesions can pose diagnostic dilemmas for both the clinician and the pathologist because it is often difficult to categorize the tumour as primary or secondary and to determine the site of origin. We present the case of a thirty year old female who was diagnosed with small cell neuro-endocrine carcinoma in the mammary gland, probably of pulmonary origin. The diagnostic challenges posed by such a case are highlighted.

## Introduction

Metastatic tumors in the breast from extra-mammary primary sites are uncommon lesions [1, 2]. This category of breast diseases constitutes about 2% of all breast malignancies [3], though the incidence rises if hematopoeitic malignancies (lymphomas and leukemias) are included [4]. The incidence is 5 - 6 times higher in females [1]. Metastasis to the mammary gland from pulmonary small cell neuroendocrine carcinoma is a rare entity [5-7] and poses a diagnostic challenge, especially if it presents as an isolated metastatic focus. The difficulty lies in differentiating it from a primary mammary gland small cell carcinoma, as well as in excluding other breast malignancies. The diagnosis therefore requires a combination of clinical and morphologic details. It is essential to establish whether the neoplasm is of primary origin or extra-mammary in nature, because the treatment and prognosis are influenced by this decision.

In this context, we present herewith the case of a young female diagnosed with a metastatic pulmonary small cell carcinoma in the left breast. The diagnostic difficulties posed by such a case are discussed in detail, along with an overview of recent research on small cell neuroendocrine carcinoma.

## Case Report

A thirty years old female patient presented in the outpatient section of the Department of Tuberculosis and Chest Diseases of our hospital with a history of fever and progressive chest pain since three months and cough with expectoration since five days. The patient was very ill and was admitted for investigation and treatment.

On clinical assessment, there was decreased air entry and dullness on percussion on the left side of the chest. Local examination revealed a 3 x 3 cm lump in the upper and outer quadrant of the left breast. The lump was firm, well defined and mobile. No lymphnodes were palpable. There were no other abnormalities.

A chest X-ray was done which revealed a homogenous opacity obscuring the entire left lung field, suggestive of a left-sided pleural effusion. Fine needle aspiration was performed on the breast lump, along with a pleural tap.

Cytospin preparations of the pleural fluid showed red blood cells and lymphocytes in the background, along with a few small groups of cells with scant cytoplasm and round, faceted nuclei with evenly distributed chromatin. The tumor cells exhibited prominent nuclear moulding.

The aspirate from the breast lump was highly cellular with sheets and clusters of cells amid scattered fat vacuoles. Numerous stippled nuclei with marked nuclear streaking and distortion were present. Nuclear moulding and faceting was a notable feature of the tumour cells. No normal ductal cells were seen in the smears.

Based on the above microscopic findings and correlating with the clinical presentation, the diagnosis of a metastatic small cell carcinoma in the breast with a probable origin from a primary focus in the lung was rendered.

Due to the effusion which obscured the lung field, the presence of a lung mass could not be confirmed. Unfortunately, the patient expired before further investigations could be carried out for confirmation of the diagnosis and detection of possible metastases to other sites.

**Figure 1:**
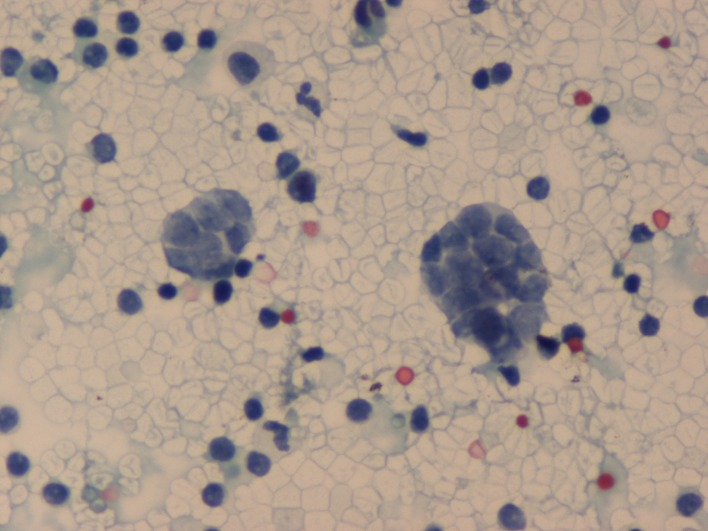
Pleural fluid – Cluster of malignant cells with scant cytoplasm, granular chromatin and nuclear faceting (PAP, ×125).

**Figure 2:**
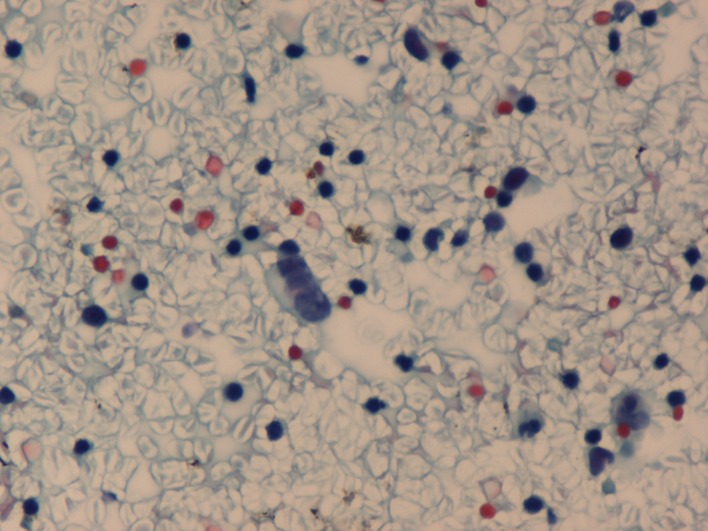
Small cell carcinoma in pleural fluid with linear arrangement of malignant cells (PAP, ×125).

**Figure 3:**
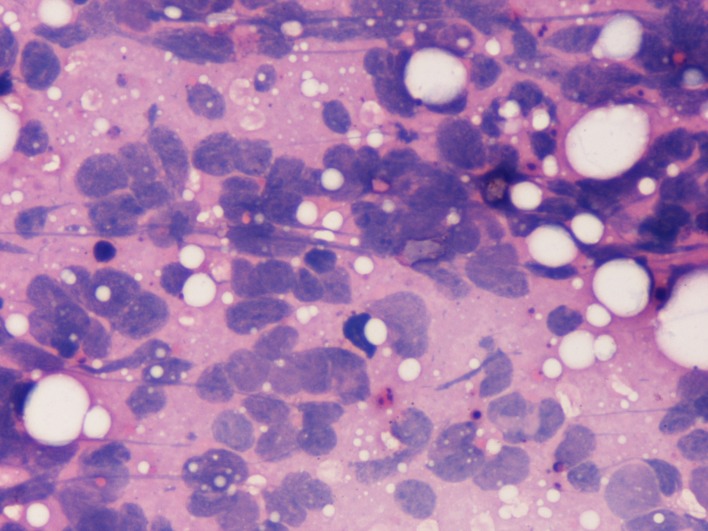
Aspirate from breast lump showing prominent nuclear moulding and streaking in malignant cells (H&E, ×500).

## Discussion

The common sources of mammary gland metastasis are lymphomas and leukemias, malignant melanoma, carcinoma lung, ovary, and kidney [2].^)^ It is imperative to correctly type the disease and identify the primary site, because, as stated earlier, the diagnosis guides the treatment protocol. The treatment of a small carcinoma remains the same regardless of whether it originates in the lung or the breast. However, the treatment of a ductal / lobular carcinoma of the breast is entirely different. With reference to the case under discussion, the crux of the problem is whether the tumor arose in the lung and metastasized to the breast or vice-versa.

Certain clinical points of good practice, if kept in mind, are helpful in such a situation. There may be a preceding history suggestive of an extra-mammary origin, (such as cough, chest pain or dyspnoea) before the breast lump was noticed. The chest x-ray will usually reveal a lung mass which can be cytologically evaluated.

It has been documented that metastases to the breast are usually well circumscribed, mobile masses, often located in the upper and outer quadrant [4, 8]. The presence of multiple nodules also goes in favor of metastasis [9]. On mammography, the mass lacks the irregular borders and micro-calcification evident in a primary carcinoma [10]. This, however, can cause confusion with a benign neoplasm.

On microscopic evaluation, the morphology of small cell carcinoma remains the same irrespective of the site of origin. A primary breast small cell carcinoma resembles its lung counterpart with regard to microscopic features and immunochemistry [10]. However, in order to differentiate a metastatic malignancy from a primary ductal or lobular carcinoma, it is advisable to look for an in-situ component, although primary mammary small cell carcinoma can also be associated with in-situ foci [11]. Significant histological features which mark the tumor as a metastatic malignancy include atypical histology for a primary lesion, and absence of an intraductal carcinoma [3]. The surrounding benign breast parenchyma shows little or no hyperplastic features [11].

On cytology, small cell carcinoma has to be differentiated from invasive lobular carcinoma, a carcinoid tumor and a malignant lymphoma (small cell type).

The tumor cells in infiltrating lobular carcinoma resemble those of small cell carcinoma with respect to cell size and scant cytoplasm. Moulding may also be present. Helpful cytological features include low cellularity, intracytoplasmic vacuoles and small nuclei and signet-ring cells [12].

The cell population in malignant lymphoma is usually dispersed rather than in clusters. The cytoplasm is intact though bare nuclei are commonly present. Nuclear moulding and streaking is not observed in malignant lymphoma. The presence of lymphoid globules is an important clue to the origin of the tumor [13].

Carcinoid tumor cells show the typical neuroendocrine nuclear features with stippled granular chromatin pattern and inconspicuous nucleoli [14]. The cells are arranged in loosely cohesive groups with dispersed cells in the background [14]. The intact cytoplasm and absence of nuclear streaking is again helpful in the differentiation from a small cell neuroendocrine carcinoma.

Traditional markers of neuroendocrine origin include NSE (neuron-specific enolase), chromogranin A, and synaptophysin. Antibodies to CD56 (Neural cell adhesion molecule) are also very helpful in differentiating small cell neuroendocrine carcinoma from non-neuroendocrine tumours [15]. However, no specific marker has yet been identified which can indicate the site of origin of the tumor. Moreover, the pattern of neuroendocrine marker expression is inconsistent in primary breast small cell carcinoma [16].

As an aid to the differential diagnosis, it has been demonstrated that while pulmonary small cell carcinomas are negative for CAM 5.2 and cytokeratin 7, primary breast small cell carcinoma shows at least focal positivity for these two markers [16].

It has been proposed that small cell carcinomas also show specific profiles of allelic loss of tumor suppressor genes which is partly dependent on primary site of origin of the tumor [17]. This has interesting implications as in future, it may be helpful in determining the site of origin. Another study has shown that a recurrent pattern of genetic changes are seen in primary small cell carcinomas of the lung and in their metastatic cells [18]. These include deletions of chromosomes 3p, 4q, 5q, 10q, 13q, 17p and DNA (deoxyribonucleic acid) over-representation on chromosomes 3q and 5p [18]. Once again, this may have a supplementary diagnostic application in the future.

The above case discussion is an attempt at highlighting the difficulties in differentiating a pulmonary small cell neuroendocrine carcinoma from an extra-pulmonary carcinoma and in identifying the site of origin. A detailed clinical history, thorough clinical examination and appropriate investigations are essential. In addition to routine diagnostic techniques, immunohistochemistry and in future, gene analysis can be of value in the differentiation and confirmation of diagnosis.
